# Safety evaluation of pretomanid, an anti-tuberculosis drug, for the treatment of pregnant and lactating women

**DOI:** 10.1128/aac.00343-26

**Published:** 2026-07-10

**Authors:** R. Bruning-Barry, J. L. Ambroso, X. Yang, D. Hickman, A. M. Harkins, M. Noble, M. Paris, S. Foraida, E. Sun, M. Beumont

**Affiliations:** 1 RTI International, Durham, North Carolina, USA; 2 Global Alliance for TB Drug Development, New York, New York, USA; Queen Mary University of London, London, United Kingdom

**Keywords:** pretomanid, tuberculosis, pregnant and lactating women

## Abstract

Safe and effective treatment options for tuberculosis (TB) are urgently needed for pregnant and lactating women due to the adverse maternal and neonatal outcomes associated with TB disease. Pretomanid is recommended by the World Health Organization for treatment of multidrug-resistant pulmonary TB in combination with bedaquiline and linezolid. However, this regimen is not recommended during pregnancy or breastfeeding due to the absence of clinical safety and efficacy data of pretomanid in these populations. This manuscript presents the nonclinical developmental and reproductive toxicology profile of pretomanid and clinical outcomes of pregnancies in women known to have been exposed to pretomanid. In nonclinical studies, pretomanid did not have a direct effect on fertility and reproductive function in female rats, and no adverse maternal or developmental effects were seen in rats or rabbits up to two times the clinical exposure during organogenesis. Additionally, no adverse developmental effects were observed in pups of pregnant rats orally dosed throughout gestation and lactation at approximately 1.5-fold clinical exposure. In the rat pre- and postnatal development study at 4.4-fold clinical exposure, there was maternal toxicity, lower pup body weights, and a delay in attaining the righting reflex, but no effects on fertility were seen in the male or female rat offspring. Clinically, 11 women became pregnant during pretomanid treatment: 5 had healthy babies, 5 had elective abortions, and 1 had a spontaneous abortion. The weight of evidence from preclinical studies and limited clinical outcomes supports further evaluation of pretomanid use in pregnant or lactating women.

**CLINICAL TRIALS:**

This study is registered with ClinicalTrials.gov as NCT03086486.

## INTRODUCTION

In 2024, an estimated 10.7 million people were diagnosed with tuberculosis (TB) worldwide, which included approximately 5.8 million men, 3.7 million women, and 1.2 million children (1). In the first updated global estimate of TB incidence during pregnancy and the postpartum period in over a decade, an estimated 239,500 TB cases occurred during pregnancy and 97,600 during the postpartum period in 2023 (2). Together, these cases accounted for approximately 9% of TB incidence among women aged 15 years and older globally, underscoring the substantial burden of TB disease in pregnant and postpartum women (2). There is a paucity of information specific to lactating women (1).

TB disease during pregnancy has been associated with adverse outcomes for the mother and neonate, including maternal anemia, need for cesarean delivery, preterm birth, low birth weight, birth asphyxia, and perinatal infant death (3, 4). In addition, pregnancy is a risk factor for TB disease, especially in the months that follow delivery (5). Despite the adverse outcomes of TB for pregnant women and neonates, pharmaceutical industry standards have historically excluded pregnant women from clinical trials of TB treatments, primarily to avoid any risk of developmental toxicity to the fetus. This has led to significant gaps in the understanding of the safety and efficacy of various therapeutic options for TB treatment in pregnant and breastfeeding women. The limited clinical data typically gathered from *ad hoc* reports during postmarketing surveillance have resulted in considerable delays in the deployment of shorter, safer, and more affordable regimens for use during pregnancy and lactation. Consequently, clinicians are often left with limited or no data to make well-informed decisions on safe and effective TB treatment for pregnant and breastfeeding women (6).

In 2022, the World Health Organization (WHO) recommended the first all-oral 6-month regimen of bedaquiline, pretomanid, linezolid, and moxifloxacin (BPaL and M if fluoroquinolone susceptibility is presumed or documented) for the treatment of rifampicin-resistant TB (RR-TB) and multi-drug-resistant TB (MDR-TB) (7). However, until June 2024, the recommended treatment for pregnant women with MDR-TB remained a 9-month or longer regimen comprising bedaquiline, levofloxacin or moxifloxacin, clofazimine, linezolid, ethambutol, high-dose isoniazid, and pyrazinamide. In June 2024, the WHO issued a rapid communication that expanded guidance to include alternative 6- to 9-month regimens for possible use in pregnancy, informed by the enrollment and retention of a limited number of pregnant women in the BEAT-Tuberculosis, endTB, and Strengthening Evidence on Optimal Treatment of Multidrug-Resistant Tuberculosis (STEM-TB) clinical trials (8, 9). The BEAT-Tuberculosis clinical trial studied a 6-month, five-drug, all-oral regimen consisting of bedaquiline, delamanid, linezolid (600 mg), levofloxacin, and clofazimine (BDLLfxC) and enrolled 10 pregnant women, of which 6 were given the standard of care, and 4 were given the experimental BDLLfxC regimen (10). The endTB observational study followed participants treated with the following all-oral 9-month regimens: BLMZ, BLLfxCZ, and BDLLfxZ. The STEM-TB cohort extended the endTB observational study in three countries. In the endTB and STEM-TB cohorts, there were 31 continued pregnancies (with one set of twins) during treatment, resulting in 32 birth outcomes, which resulted in 26 live births (81%), 2 spontaneous abortions, and 4 for whom there was no information (8).

There is still a pressing need for alternate regimens that offer simpler and/or more affordable treatment options for pregnant and lactating women with RR/MDR-TB. The BPaL/M regimen is the first-line recommended regimen for the general population with RR/MDR-TB but is not recommended for pregnant and lactating women (11). The aim of this manuscript is to summarize the preclinical developmental and reproductive toxicity and clinical pregnancy data available for pretomanid toward future consideration of pretomanid-containing regimens for use in clinical research and treatment of pregnant and lactating women.

## MATERIALS AND METHODS

### Nonclinical studies

All animal studies were conducted in compliance with US Food and Drug Administration Good Laboratory Practice (GLP) regulations in test facilities that were accredited by the Association of Assessment and Accreditation of Laboratory Animal Care.

### Test article and formulations

Pretomanid, (6 *S*)-2-nitro-6-{[4-(trifluoromethoxy)benzyl]oxy}-6,7-dihydro-5*H*-imidazo[2,1-]*b* [1,3] oxazine, was synthesized by Metrics and Mayne Pharma Inc. (≥99.6% purity by high-performance liquid chromatography [HPLC] area%). The formulations used in the preclinical development and reproductive toxicology studies were suspensions either in 0.4% or 0.5% (wt/vol) sodium carboxymethylcellulose (Sigma-Aldrich [Batch/Lot No. SLBQ0706V or 055K0156]) in deionized water. Dosing was oral by gavage, and formulations were analyzed to confirm drug concentrations.

### Animals and husbandry

Male and female Sprague-Dawley rats (Charles River Laboratories) and female New Zealand White rabbits (Covance Research Products) were used in these studies. Animals were group housed, except when pregnant or paired for mating. Rats were provided Certified Rodent Diet #5002 (PMI Nutrition International). Rabbits were provided Certified Rabbit Chow #5322 (PMI Nutrition International) *ad libitum*. Drinking water purified by reverse osmosis was provided *ad libitum*. Environmental conditions across studies were set to maintain relative humidity at 30%–70% and temperature of 64°F–79°F and 61°F–72°F for rats and rabbits, respectively. Room lighting was set to provide a 12-h light/dark cycle.

### Experimental designs

Standard developmental and reproductive toxicology studies, including fertility and early embryonic development (FEED) in rats, embryo-fetal development (EFD) in rats and rabbits, and pre- and postnatal development (PPND) in rats, were conducted with pretomanid according to the International Council for Harmonisation of Technical Requirements for Human Use (ICH) S5(R3) and M3(R2) guidance documents for industry (12, 13). Although both male and female rats were evaluated in the FEED study described in this manuscript, the primary focus of this risk assessment is on pregnant and lactating women, fetuses, and neonates. Detailed male reproductive findings will be reported separately in a focused manuscript describing the testicular findings in rodent toxicology studies with pretomanid.

### Dose level selection

Dose selection was guided by ICH S5(R3) guidance for industry, which provides recommendations for dose level selection in developmental and reproductive toxicology studies (12). For this program, toxicity-based endpoints were dose-limiting and were used to define the high dose. This approach reflects the narrow safety margins typical of antibiotics, where clinically relevant exposures often occur close to doses associated with toxicity in nonclinical species. Although it is generally desirable to establish a no-observed-adverse-effect level (NOAEL) or safe dose level with large multiples (e.g., 10×) over clinical exposure, antibiotics frequently yield NOAELs at exposures near or below those required for clinical efficacy.

Dose levels for the rat FEED study were informed by a 13-week general toxicity study in rats administered pretomanid at 0, 10, 30, 100, or 300 mg/kg of body weight/day, in which the NOAEL was 30 mg/kg of body weight/day for both sexes. Histologic effects in male reproductive organs were observed for pretomanid; therefore, in the FEED study, the pre-cohabitation dosing interval was extended to 70 days (10 weeks) to encompass all stages of spermatogenesis, resulting in a total male dosing duration comparable to the 13-week toxicity study. In the 13-week study, body weight and reproductive organ weights were reduced at 100 and 300 mg/kg of body weight/day, with additional organ weight changes observed at ≥30 mg/kg of body weight/day. Females were dosed for 2 weeks prior to cohabitation, and 4-week toxicity data from the 13-week study were used to inform dose selection, which is similar to the duration used to dose females in the FEED study spanning dosing during pre-mating through gestation day 6 (approximately 5–6 weeks). In the 13-week toxicity study, female body weight gain and organ weight reductions were observed at ≥100 mg/kg of body weight/day (including ovary and uterus weights at 300 mg/kg of body weight/day). Based on these findings, doses of 10, 30, and 100 mg/kg of body weight/day were selected for the FEED study. The 100 mg/kg of body weight/day dose was anticipated to impact fertility parameters for males and/or body weight gain for both sexes, 30 mg/kg of body weight/day was expected to be minimally toxic for males, and 10 mg/kg of body weight/day was selected to identify an NOAEL in both sexes.

Non-GLP dose range-finding embryo-fetal development studies were conducted in pregnant rats and rabbits to inform dose selection for the pivotal GLP EFD studies. In rats, doses of 0, 10, 30, 100, and 300 mg/kg of body weight/day were evaluated. At 300 mg/kg of body weight/day, excessive maternal toxicity was observed, including unscheduled euthanasia of two animals, adverse clinical signs, and body weight loss. At this dose, post-implantation loss and reduced fetal body weights were also observed. At 30 and 100 mg/kg of body weight/day, reduced maternal body weight gain and food consumption were observed, supporting the selection of a high dose associated with minimal maternal toxicity. Fetal body weights were also reduced at 100 mg/kg of body weight/day. A low dose of 10 mg/kg of body weight/day was selected to identify an NOAEL.

In non-GLP dose-range-finding EFD studies in rabbits, pretomanid doses of 0, 10, 30, 100, and 300 mg/kg of body weight/day were evaluated. The 300 mg/kg of body weight/day dose was not tolerated, with multiple animals dying or requiring early termination. Maternal body weight loss occurred at 100 mg/kg of body weight, and reduced body weight gain was observed at 30 mg/kg of body weight/day. Decreased food consumption and reduced fecal output were observed at ≥30 mg/kg of body weight/day, with slight reductions in fetal body weights at 100 mg/kg of body weight/day. Based on these findings, doses of 0, 10, 30, and 60 mg/kg of body weight/day were selected for the GLP EFD study, with 60 mg/kg of body weight/day anticipated to produce minimal maternal toxicity and 10 mg/kg of body weight/day to define an NOAEL.

Dose levels for the PPND study were selected based on the results of the EFD study. Because maternal effects at 100 mg/kg of body weight/day exceeded criteria used to define minimal maternal toxicity, the proposed high dose level was 60 mg/kg of body weight/day, which was above the NOAEL in the 13-week rat general toxicity study. The mid- and low-dose levels were selected using half-log spacing (20 and 7 mg/kg of body weight/day, respectively), with the low dose anticipated to be a safe exposure.

#### Rat FEED study

Pretomanid or vehicle was administered by oral gavage to male rats (25/group) once daily beginning 70 days prior to cohabitation and continuing through a 3-week period of cohabitation to day 98 of the study. Female rats (25/group) received the test article or vehicle once daily beginning 15 days before cohabitation (maximum 17 days) and continuing through day 7 of presumed gestation day (GD) 7. Dose levels were 0 (vehicle), 10, 30, and 100 mg/kg of body weight/day. In addition, male rats (15/group) treated for 14 weeks before cohabitation were paired with untreated female rats, and male rats treated for 14 weeks and then allowed to recover for 10 weeks before cohabitation were paired with untreated females. The following parameters were evaluated: viability, clinical observations, body weights, body weight gains, feed consumption, estrous cyclicity (female rats), cesarean-sectioning observations (female rats), necropsy observations, histopathology evaluation of epididymides, prostate, and seminal vesicles (male rats), blood hormone analysis (inhibin B, follicle-stimulating hormone, and testosterone levels in male rats), and sperm concentration and motility (male rats). On GD13, female rats were sacrificed via carbon dioxide asphyxiation and examined for pregnancy status.

#### Rat EFD study

Eighty healthy, mated female rats were randomly assigned to four groups of 20 rats/group. Forty-one additional rats were assigned: 5 in vehicle control and 12 each in pretomanid-treated groups for toxicokinetic (TK) sample collection. Pretomanid was administered orally by gavage once daily on GD 7 through 17 at doses of 0, 10, 30, and 100 mg/kg of body weight/day to the main study rats. Rats assigned to the TK portion of the study were administered pretomanid or vehicle on GD 7 through 18 using the same dose and volume as main study rats. On GD 7 and 17, blood samples were obtained from each rat assigned to the TK portion of the study to define drug concentration vs time profiles. On GD 18, following dose administration, the TK rats were sacrificed at four timepoints, and maternal and fetal blood samples were collected to compare maternal and fetal TK profiles. All rats assigned to the main study were sacrificed, and cesarean section was performed on GD 21. The following parameters were evaluated: viability, clinical observations, body weights, body weight gains, feed consumption, necropsy observations, maternal and fetal TK, litter observations, fetal body weights, fetal sex, and fetal morphology (gross external, soft tissue, and skeletal examinations).

#### Rabbit EFD study

Eighty presumed pregnant New Zealand White rabbits were randomly assigned to four groups of 20 rabbits/group. An additional 18 rabbits, 3 in vehicle control and 5/group in pretomanid-treated groups, were assigned to TK sample collection. Pretomanid was administered orally by gavage once daily on GD 7 through 19 at doses of 0, 10, 30, and 60 mg/kg of body weight/day to the main study rabbits. Rabbits assigned to the TK portion of the study were administered pretomanid or vehicle on GD 7 through 20 using the identical regimen. On GD 7 and 19, blood samples were collected from each rabbit assigned to the TK satellite groups (three timepoints from controls and seven timepoints from drug-treated groups). To compare maternal and fetal TK profiles, on GD 20, following the last dose, blood samples were collected from one pregnant rabbit and pooled fetal blood samples at one timepoint in controls and five timepoints in drug-treated groups. The resulting plasma samples were analyzed for concentrations of pretomanid. All rabbits assigned to the main study were sacrificed and cesarean sectioned on GD 29. The following parameters were evaluated: viability, clinical observations, body weights, body weight gains, feed consumption, necropsy observations, maternal and fetal TK, litter observations, fetal body weights, fetal sex, and fetal morphologic evaluations (gross external, soft tissue, and skeletal).

#### Rat PPND study

Pregnant Sprague-Dawley rats (F0) were assigned to four groups of 25 rats/group. An additional 16 rats (4/group) were assigned to the TK sample collection. Pretomanid was administered once daily by oral gavage from GD 7 through lactation day (LD) 20 at doses of 0, 7, 20, and 60 mg/kg of body weight/day to main study rats. Rats assigned to the TK portion of the study were administered pretomanid or vehicle using an identical regimen. Finally, 25 rats/sex/group from the F1 generation (offspring resulting from the first cross of the F0 generation) were selected for further evaluation as described below. Observations of the F0 animals included clinical signs, body weight, and feed consumption during gestation, parturition, and litter data, success in rearing offspring to weaning, and anatomical pathology. Analysis of plasma levels of pretomanid was conducted for F0 females (LD 14 and LD 20) and F1 pups (LD 14). Assessment of maternal milk levels for pretomanid was conducted for F0 females (LD 14). Observations of the offspring (F1) included survival, sex, body weights, gross abnormalities, and physical development. Following weaning, selected F1 animals were observed for clinical signs, body weights, and anatomical pathology. The selected F1 animals were also evaluated for growth, sexual maturation, motor activity, behavioral testing (passive avoidance and Morris water swim maze), reproductive performance, fertility, parturition, and F2 litter data (LD 0, birth). Observations of the offspring (F2) at birth (LD 0) included viability, clinical signs, sex, body weights, and gross abnormalities.

### Bioanalysis

Concentrations of pretomanid in plasma and milk were measured using HPLC with tandem mass spectrometry detection. In the rat EFD study, blood samples were collected from the lateral tail vein. In the rabbit EFD study, blood samples were collected from the medial auricular artery of each rabbit assigned to the TK group. In the PPND study, blood samples were collected from all F0 females and TK F1 pups via the sublingual vein (F0 females) or abdominal vena cava after intraperitoneal injection of euthanasia solution (F1 pups). Milk samples (maximum obtainable volume) were collected from all four TK F0 females per group after isoflurane inhalation for the determination of milk concentrations.

### Margin of safety calculation

The approved dose of pretomanid in the BPaL regimen is 200 mg/day, which produces a median steady-state *C*_max_ of 3.2 µg/mL and AUC_0–24 h_ of 58.8 h·µg/mL (14). These exposures were compared to those in animal reproductive toxicology studies at the NOAEL to calculate the margin of safety.

### Clinical studies

A search for pretomanid pregnancy-related cases was conducted using the TB Alliance safety database. Relevant Preferred Terms within Medical Dictionary for Regulatory Activities were selected, and search results were reviewed. The complications during and after the pregnancy and newborn/infant outcomes were assessed. Cases were classified as “exposure during pregnancy” if the pregnancy occurred from the first day of treatment up to 30 days after the end of treatment with pretomanid.

## RESULTS

### Nonclinical reproductive toxicology studies

In the rat FEED study (Table 1), male rats tolerated daily oral doses of pretomanid at 10, 30, or 100 mg/kg of body weight for 13–14 weeks, albeit with clinical signs of ill health (urine-stained fur, salivation, and dehydration), reduced body weights (82% of control), and lower feed consumption (~90% of control) at 100 mg/kg of body weight/day. The fertility index (number of pregnancies/number of rats that mated) was reduced at 30 mg/kg of body weight/day (64% vs 92% in control), and complete infertility (fertility index, 0%) was observed at 100 mg/kg of body weight/day when treated males were paired with treated females at exposures estimated to be approximately one and four times the approved clinical dose of pretomanid (200 mg/day), respectively. At 100 mg/kg of body weight/day, males also had diffuse atrophy of the spermatogenic cells in testes, an absence of spermatozoa in the epididymides, visibly smaller testes and epididymides, lower mean serum inhibin B concentration (below the lower limit of quantitation of 75 pg/mL in all 25 male rats compared with 13 of 25 for male controls), and higher serum follicle-stimulating hormone concentration (mean of 17.5 ng/mL compared with 8.8 ng/mL for male controls). When dosing stopped, clinical signs of diminished body weight gain improved; however, at the end of a 10-week recovery period, adult males that had been given pretomanid at 100 mg/kg of body weight/day were still infertile and had no microscopic evidence of spermatogenesis. Fertility did improve somewhat over the recovery period in males that had been given pretomanid at 30 mg/kg of body weight/day (fertility index after recovery of 78.6% vs 86.7% in controls), where the exposure (AUC_0–24 h_) was approximately 1.4 times the clinical exposure at the approved clinical pretomanid dose, suggesting that testicular effects were at least partially reversible at this dose level.

**TABLE 1:** Summary of body weight, food consumption, fertility, and mid-gestation uterine examination data from the pretomanid fertility and early embryonic development study in rats^*a*^^,*b*,^^*c*^^*,^g^*^

	Dose (mg/kg of body weight/day)			
	0	10	30	100
Number of females/ males	25/25	25/25	25/25	25/25
Male day 98 body weight (g)	600.4 ± 49.3	597.1 ± 45.9	586.9 ± 70.6	490.9 ± 45.2^*f*^
Male recovery day 193 body weight (g)	695.5 ± 65.3	699.4 ± 63.6	707.4 ± 97.8	645.1 ± 61.1
Male food consumption (g/day)				
Premating days 1–70	29.5 ± 2.4	28.9 ± 2.7	30.1 ± 2.5	26.3 ± 3.4^*f*^
Mating days 85–92	28.0 ± 2.8	28.4 ± 3.1	29.2 ± 2.2	26.8 ± 3.6
Female body weight (g), day prior to cohabitation	256.2 ± 15.7	251.9 ± 15.5	249.2 ± 17.7	232.6 ± 12.9^*f*^
Female food consumption (g/day)				
Premating days 1–8	18.7 ± 2.2	17.8 ± 1.7	18.3 ± 3.8	10.1 ± 3.2^*f*^
Gestation days 0–8^*d*^	23.2 ± 2.1	22.5 ± 2.9	22.7 ± 2.6	Non-pregnant
Gestation days 8–13	24.5 ± 2.3	24.6 ± 3.0	26.7 ± 2.6^*e*^	Non-pregnant
Fertility data from pairing males after 70 days of treatment with treated females				
Estrous stages/14 days	3.2 ± 0.5	3.1 ± 0.5	3.3 ± 0.7	2.6 ± 0.9^*f*^
Days in cohabitation	2.1 ± 0.9	2.1 ± 2.8	2.0 ± 1.0	3.2 ± 1.7^*e*^
Mating index (%)	100	95.8	100	100
Fertility index (%)	92.0	95.6	64.0	0.0^*f*^
Number of pregnant females	23	24	16	0
Uterine examination data				
Corpora lutea	15.6 ± 2.9	16.4 ± 3.1	15.0 ± 2.1	Non-pregnant
Implantation sites	14.6 ± 2.3	15.0 ± 1.9	13.3 ± 4.6	Non-pregnant
Live embryos	13.8 ± 2.3	14.1 ± 2.5	12.1 ± 4.4	Non-pregnant
Resorptions	0.8 ± 1.0	0.9 ± 1.2	1.2 ± 1.0	Non-pregnant
Placentae appeared normal (%)	23 (100)	23 (95.8)	16 (100)	Non-pregnant
Fertility data from pairing males after 98 days of treatment with untreated females				
Days in cohabitation (days)	2.7 ± 0.7	3.0 ± 1.3	3.5 ± 1.4	3.0 ± 1.3
Fertility index (%)	100.0	92.3	64.0	7.1^*f*^
Number of pregnant females	15	12	9	1^*f*^
Uterine examination data				
Corpora lutea	15.9 ± 1.4	14.9 ± 1.6	13.8 ± 2.0^*e*^	12.0
Implantation sites	15.0 ± 1.6	14.6 ± 1.2	11.9 ± 4.3	1.0
Live embryos	14.5 ± 1.9	13.3 ± 1.5	10.8 ± 3.9^*e*^	
Placentae appeared normal (%)	15 (100)	12 (100)	9 (100)	
Fertility data from pairing treated males after a 10-week recovery (day 161) with untreated females				
Days in cohabitation (days)	1.7 ± 0.8	2.1 ± 1.4	1.6 ± 0.7	3.9 ± 2.6
Fertility index (%)	86.7	92.8	78.6	0.0^*f*^
Number of pregnant females	14	14	11	0
Uterine examination data				
Corpora lutea	14.8 ± 1.7	15.3 ± 3.4	16.4 ± 1.4	
Implantation sites	14.7 ± 1.6	14.1 ± 4.1	15.7 ± 1.5	
Live embryos	14.4 ± 1.6	13.6 ± 4.2	15.1 ± 1.5	
Placentae appeared normal (%)	14 (100)	14 (100)	11 (100)	

^*a*^ Data are presented as mean per group ± standard deviation.

^*b*^ Mating index is the number of mating pairs with confirmed mating/the number of mating pairs cohabitated.

^*c*^ Fertility index is the number of mating pairs with a pregnant female/the number of mating pairs cohabitated.

^*d*^ Dosing occurred through day 7 of gestation.

^*e*^ Significantly different than the corresponding vehicle control, *P* ≤ 0.05.

^*f*^ Significantly different than the corresponding vehicle control, *P* ≤ 0.01.

^*g*^ The study was extended by 10 weeks to allow a recovery assessment in 15 males/group. These male rats were bred with untreated (naïve) females during the week following termination of the treatment phase (day 98) and 10 weeks later (days 161–175).

There were no effects of pretomanid in adult male rats treated for 13 weeks at 10 mg/kg of body weight/day, which is estimated to be approximately half of the human exposure for the approved clinical dose of pretomanid. The male rat fertility findings align with observations from adult mice and rats in the general toxicology program (15), where testicular atrophy was observed in rats as soon as 2 days after dosing with a high dose (500 mg/kg of body weight/day) and as low a dose as 30 mg/kg of body weight/day in a 26-week rat study. In contrast, no testicular histologic effects were observed in monkey toxicity studies at doses up to 1,000 mg/kg of body weight/day and durations up to 39 weeks, including studies of 13- and 39-week durations in sexually mature monkeys (16).

The rat fertility data were essentially the same when treated or untreated females were mated with treated males, and therefore, the findings were attributed to pretomanid’s effects on male testes/spermatogenesis and not considered a direct effect on reproductive function in female rats (Table 1). Female rats tolerated daily oral doses of pretomanid at 10, 30, or 100 mg/kg of body weight for 2 weeks, albeit with clinical signs of ill health, body weight loss (up to 91% of controls on day 15 pre-cohabitation), reduced feed consumption (to 71% of controls during pre-cohabitation), reduced number of estrous stages per 14 days (2.6 cycles vs 3.2 cycles in controls), and a greater incidence of prolonged diestrus at 100 mg/kg of body weight/day. The effects on estrus cycling were most likely secondary to reduced feed consumption and weight loss rather than a direct effect of pretomanid (17). When paired with treated males, the fertility index was reduced at ≥30 mg/kg of body weight/day (to 64%) for both treated and untreated females. At 100 mg/kg of body weight/day, the number of days in cohabitation was higher, and there was complete infertility. Reduced fertility was almost certainly due to the effects on spermatogenesis in the treated adult males; however, the longer period in cohabitation may potentially be the consequence of the reduced estrus cycling in the high-dose females.

In the rat EFD study (Table 2), maternal effects of pretomanid at ≥30 mg/kg of body weight/day included lower maternal body weights (82% of controls on GD17) and/or body weight gain and lower feed consumption (70% of controls) throughout the entire dosing period. Body weight gains at 30 and 100 mg/kg of body weight/day were significantly reduced for the entire treatment period (GD 7 through 18; 16% and 77% less than controls) but recovered and exceeded control group values during the post-dose period. Mean body weights on GD 21 in the 30 and 100 mg/kg of body weight/day groups were 98% and 85% of the control group, respectively. Absolute feed consumption for the entire treatment period was 90% and 60% for the 30 and 100 mg/kg of body weight/day groups compared with the control group, respectively. Clinical signs, including fur staining and mild dehydration, were observed at 100 mg/kg of body weight/day. Developmental effects included increased early resorptions/litter (1.8 compared with 0.7 resorptions/litter in controls) and lower fetal body weights (91% of controls) at 100 mg/kg of body weight/day. Fetal body weights were within the ranges observed historically at the testing facility. No external, soft-tissue, or skeletal malformations or variations were caused by doses of pretomanid as high as 100 mg/kg of body weight/day. The maternal NOAEL for pretomanid was 10 mg/kg of body weight/day based on reduced maternal body weight and/or body weight gain and feed consumption during the entire dosage period at ≥30 mg/kg of body weight/day. The NOAEL for effects on fetal development was 30 mg/kg of body weight/day based on increased early resorptions and lower fetal body weights at 100 mg/kg of body weight/day. The NOAEL for fetal development occurred at maternal exposures approximately two times the exposure at the approved clinical dose of pretomanid (Table 3). Pretomanid was not teratogenic or a selective developmental toxicant, as no malformations were observed, and fetal effects occurred only at dose levels that produced maternal toxicity.

**TABLE 2:** Mean maternal and fetal data from the rat embryo-fetal development study with pretomanid^*c*^

Maternal dose (mg/kg of body weight/day)				
	0	10	30	100
Number of pregnant dams (GD 21)	18	18	19	19
Maternal body weight (GD 17; g)	342.8	351.5	330.9	281.0^*b*^
Maternal body weight (% control)		102.5	96.5	82.0^*b*^
Maternal body weight gain (GD 7–18, g)	87.8	90.2	73.8^*b*^	19.9^*b*^
Maternal food consumption (GD 7–18; g/day)	28.0	28.1	25.1^*b*^	16.8^*b*^
Maternal food consumption (% control)		100.4	89.6^*b*^	60.0^*b*^
Corpora lutea	15.7	16.4	15.7	15.0
Implantation sites	14.6	15.6	15.2	14.4
Pre-implantation loss (%)	6.8	4.6	2.9	3.7
Post-implantation loss (%)	4.6	3.8	3.5	8.6^*a*^
Fetal alteration summary				
Litters evaluated (*N*)	18	18	19	18
Fetuses evaluated (*N*)	249	271	279	240
Live fetuses/litter	13.8	15.0	14.7	12.6
Sex ratio (% male)	56.2	47.4	56.2	49.4
Fetal weight (g, sexes combined)	5.51	5.58	5.34	5.01^*b*^
Litters with any alteration, *N* (%)	8 (44.4)	4 (22.2)	8 (42.1)	6 (33.3)
Fetuses with any alteration, *N* (%)	13 (5.2)	4 (1.5)	10 (3.6)	9 (3.8)
Fetuses with any alteration/litter mean ± SD (%)	5.2 ± 6.8	1.5 ± 3.0	3.4 ± 4.6	4.1 ± 7.1
Fetal ossification sites—hindlimb phalanges, mean ± SD (per fetus/litter)	6.87 ± 1.26	6.61 ± 0.95	6.37 ± 1.12	5.74 ± 0.80^*b*^

^*a*^ One dam at 100 mg/kg of body weight/day had a litter consisting of all early resorptions; excluded from group mean.

^*b*^ Significantly different than the corresponding vehicle control, *P* ≤ 0.01.

^*c*^ GD, gestation day.

**TABLE 3:** Mean maternal and fetal systemic exposure in the rat and rabbit embryo-fetal development studies with pretomanid*^a^*^,^*^b^*

Study	Sampling day	Dose (mg/kg of body weight/day)	*C*_max_ (µg/mL)	AUC_0–24 h_ (µg·h/mL)	Exposure margin to approved clinical dose	
					*C*_max_	AUC_0–24 h_
Rat EFD study (maternal)	GD 18	10	2.09	35.2	0.7	0.6
		30	6.28	104	2.0	1.8
		100	17.2	245	5.4	4.2
Rat EFD study (fetal)	GD 18	10	2.92	42.8	NA	NA
		30	7.64	124	NA	NA
		100	14.6	251	NA	NA
Rabbit EFD study (maternal)	GD 19	10	1.40	12.2	0.4	0.2
		30	3.63	34.1	1.1	0.6
		60	8.24	87.8	2.6	1.5
Rabbit EFD study (fetal)	GD 20	10	0.94	9.82	NA	NA
		30	2.67	39.2	NA	NA
		60	3.78	44.8	NA	NA

^*a*^ *C*_max_, peak serum concentration; AUC_0–24 h_, area under the concentration-time curve from 0 to 24 h; GD, gestation day; and NA, not applicable.

^*b*^ Exposure margins calculated based on clinical steady-state *C*_max_ at the approved clinical dose of 200 mg pretomanid and AUC_0–24 h _values of 3.2 µg/mL and 58.8 h·µg/mL, respectively ([14]; ZeNix Trial; ClinicalTrials.gov NCT03086486).

In the rabbit EFD study (Table 4), reduced maternal body weight and/or body weight gain and feed consumption occurred during the entire dosage period at ≥30 mg/kg of body weight/day. A statistically significant loss (−0.01 kg) in mean body weight occurred at the high dose of 60 mg/kg of body weight/day during the entire treatment period relative to gains (+0.24 kg) in the control group. In addition, rabbits treated with 30 mg/kg of body weight/day pretomanid gained less body weight (21% less) than rabbits in the control group during treatment. Corresponding to the mean body weight losses and reduced body weight gains, absolute feed consumption values were reduced to 91% and 58% of control values, respectively, for the entire treatment period in the 30 and 60 mg/kg of body weight/day groups. At 60 mg/kg of body weight/day, maternal females also had reduced fecal output. Despite these maternal effects, no cesarean section or fetal parameters were affected by treatment with pretomanid up to 60 mg/kg of body weight/day. The maternal NOAEL for pretomanid was 10 mg/kg of body weight/day based on reduced maternal body weight and/or body weight gain and feed consumption during the entire dosage period at ≥30 mg/kg of body weight/day. Fetal growth (body weight) was not affected, and no gross external, soft tissue, or skeletal fetal alterations (malformations or variations) were associated with pretomanid treatment up to 60 mg/kg of body weight/day. The NOAEL for effects on fetal development was 60 mg/kg of body weight/day, which produced average maternal exposures in rabbits approximately two times the exposure at the approved clinical dose of pretomanid.

**TABLE 4:** Mean maternal and fetal data from the rabbit embryo-fetal development study with pretomanid^*c*^

	Dose (mg/kg of body weight/day)			
	0	10	30	60
Number of pregnant does	19	19	20	19
Maternal body weight (GD 19; kg)	3.57	3.58	3.52	3.34^*a*^
Maternal body weight (% control)		100.3	99.2	93.6^*a*^
Maternal body weight gain (GD 7–20, kg)	+0.24	+0.26	+0.19	−0.01^*b*^
Maternal food consumption (GD 7–20; g/day)	161.1	158.0	146.8	93.5^*b*^
Maternal food consumption (% control)		98.1	91.1	58.0^*b*^
Corpora lutea	8.8	9.6	9.0	9.5
Implantation sites	8.3	9.2	8.8	9.1
Pre-implantation loss (%)	5.8	4.4	4.0	3.7
Post-implantation loss (%)	2.8	0.6	2.9	3.1
Fetal alteration summary				
Litters evaluated (*N*)	19	19	20	19
Fetuses evaluated (*N*)	151	174	170	167
Live fetuses/litter	7.9	9.2	8.5	8.8
Sex ratio (% male)	53.8	48.9	53.2	56.7
Fetal weight (g)	41.92	41.07	41.81	39.50
Litters with any alteration, *N* (%)	7 (36.8)	9 (47.4)	5 (25.0)	12 (63.2)
Fetuses with any alteration, *N* (%)	11 (7.3)	17 (9.8)	9 (5.3)	16 (9.6)
Fetuses with any alteration/litter mean ± SD (%)	7.4 ± 11.2	12.9 ± 23.8	5.2 ± 11.1	9.5 ± 8.9

^*a*^ Significantly different than the corresponding vehicle control, *P* ≤ 0.05.

^*b*^ Significantly different than the corresponding vehicle control, *P* ≤ 0.01.

^*c*^ GD, gestation day.

In the rat PPND study, there were no adverse developmental effects in pups of pregnant rats orally dosed with pretomanid up to 20 mg/kg of body weight/day (exposure approximately equivalent to the approved clinical dose of pretomanid) from GD 7 through LD 20 (Table 5 ). Specifically, there was no effect of pretomanid on maternal survival, clinical findings, gestation/lactation body weights, body weight changes, gestational food consumption, and parturition outcomes, or any F1 pup data to weaning, including survival, sex ratio, body weights, physical development, sexual maturation, motor activity, neurobehavioral evaluations, reproductive performance/fertility through parturition of F2 litters, and macroscopic findings for any of the F0 females and the F1 and F2 generations. At 60 mg/kg of body weight/day, which is estimated to be three times the clinical exposure and where there was maternal toxicity (lower body weights and body weight gains, lower feed consumption), there were lower pup body weights starting on LD4 continuing through postnatal day 28 (7%–10% lower than controls) that recovered post-weaning and a delay in attaining the righting reflex in the F1 generation (exposed *in utero* from implantation through parturition and during lactation). There were no other effects on physical or sexual development, reproductive performance, or fertility, unlike those observed in the FEED study in adult rats. Pretomanid concentrations in breast milk were found to be approximately 1.5 times those in maternal plasma, but plasma concentrations in F1 pups were much lower than those in maternal plasma.

**TABLE 5:** Maternal data, litter survival, and pup survival in the rat pre- and postnatal development study with pretomanid^*b*^

	Dose (mg/kg of body weight/day)			
	0	7	20	60
Females pregnant/group	25	25	25	25
Females with liveborn	25	24	25	25
Pups born, mean/litter	12.2	13.4	13.4	13.2
Mean stillborn pups/litter	0.2	0.1	0.1	0.3
Sex ratios (%)	50.21	50.87	46.71	51.29
Viability index	98.20	97.56	98.02	96.63
Lactation index	99.50	100.00	100.00	100.00
F1 pup body weights for males and females combined during lactational exposure to pretomanid (g)				
LD 4 (post-culling)	11.75	11.30	10.98	10.69**
LD 7	19.27	19.02	18.41	17.34**
LD 14	37.61	37.84	37.38	34.92*
Postnatal day 21 (first day of weaning)	61.30	61.15	59.52	56.78**
F1 male pup body weights post-weaning (g)				
Week 8 (premating)	529.5	528.8	511.0	489.9*
Week 11 (pairing)	598.8	615.9	589.4	572.8
Week 14 (postmating)	668.7	672.7	648.9	626.4
F1 female pup body weights post-weaning (g)				
Week 8 (premating)	287.7	286.6	296.8	282.5
Gestation day 0	308.7	304.6	316.8	300.4
Gestation day 20	451.9	459.4	466.7	449.1
Exposure (ng/mL)				
F_0_ maternal plasma^*a*^ (4 h post-dose, LD 20)		1,160 (0.4)^*a*^	2,840 (0.9)^*a*^	10,700 (3.4)^*a*^
F_0_ maternal milk (6 h post-dose, LD 14)		1,630	4,070	17,500
F_1_ pup plasma (4 h post-maternal dose, LD 14)		37.2	119	436

^*a*^ F0 maternal plasma data are presented as mean concentration in ng/mL (exposure margin compared to 3.2 µg/mL [14]).

^*b*^ LD, lactation day. *P < 0.05; **P < 0.01.

### Pregnancy data from clinical trials

The TB Alliance safety database identified a total of 45 reports of pregnancies arising from 10 completed or ongoing pretomanid clinical trials at the time this manuscript was written, and the details are provided in Table S1. From those 45 reports of pregnancies, only 11 women became pregnant during pretomanid treatment (defined as if the pregnancy occurred from the first day of treatment up to 30 days after the end of treatment with pretomanid) (Table 6). The mean age was 27 years (range: 20– 39 years); the last menstrual period ranged from 1 to 58 days prior to the last dose of pretomanid. As per protocol for most clinical trials, women were to discontinue treatment if pregnancy was identified during therapy. Only one woman continued treatment after a positive pregnancy test (TB-PRACTECAL, ClinicalTrials.gov number: NCT03942354) (18) while she was receiving BPaLC; exposure during pregnancy to pretomanid and its companion drugs was 58 days, and the participant delivered a healthy baby.

**TABLE 6:** Listing of women with a confirmed pregnancy classified as exposed to pretomanid in clinical trials^*b*^

Study participant age (years)	TB treatment regimen	Duration of exposure to pretomanid^*a*^ (days)	Pregnancy outcome as reported
35	PaHRZ/PaHR	27	Healthy baby
23	PaMZ	45	Elective abortion
39	PaMZ	46	Healthy baby
31	BPaMZ	45	Spontaneous abortion
31	BPaL	1	Elective abortion
30	BPaL	10	Elective abortion
20	BPaL	41	Elective abortion
20	BPaLC	58	Healthy baby
22	BPaLC	21	Healthy baby
21	BPaLM	2	Healthy baby
27	BPa sutezolid N-acetyl cysteine	19	Elective abortion

^*a*^ Exposure to pretomanid is estimated from the study day at the end of therapy treatment minus the date of the last menstrual period. Table includes data reported in reference 18.

^*b*^ H, isoniazid; R, rifampin, Z, pyrazinamide; M, moxifloxacin; L, linezolid; B, bedaquiline; Pa, pretomanid; and C, clofazimine.

Of the 11 pregnancy reports during treatment, the following were observed:

- Five women had full-term healthy babies, including the participant who completed treatment as planned. Treatment for these patients consisted of BPaLC for two and PaMZ, BPaMZ, and BPaLM for one each.
- Five women had elective abortions from the following regimens: three were from BPaL, one BPaS (where S denotes sutezolid), and one BPaMZ.
- One woman had a spontaneous abortion: a 31-year-old participant received PaMZ for approximately 30 days while pregnant, and approximately 3 weeks after completing treatment, had a miscarriage.
- No congenital malformations were reported from any pregnancies.

## DISCUSSION

### Unmet need for MDR-TB treatment in pregnancy and lactation

This manuscript presents the nonclinical reproductive and developmental toxicity data for pretomanid, together with the limited available clinical pregnancy data. MDR-TB is a serious, life-threatening disease, and active TB disease during pregnancy and the postpartum period is associated with adverse outcomes for both the mother and neonate. Shorter, simpler, and more accessible regimens like the BPaL/M regimens are urgently needed for pregnant and lactating populations, given their widespread use and benefits in non-pregnant patients. The discussion below summarizes the nonclinical reproductive and developmental toxicity findings, places them in a regulatory and clinical context, and considers the benefits and risks of further evaluating pretomanid use in pregnant and lactating women.

### Summary of nonclinical reproductive and developmental findings

A complete package of nonclinical reproductive studies has been conducted with pretomanid and collectively supports further evaluation in pregnant and lactating women.

In the FEED study, pretomanid impacted fertility in adult male rats without a direct effect on female fertility, suggesting a low risk of impacting fertility in women of childbearing potential. In EFD studies, no adverse maternal or embryo-fetal effects in rats or rabbits dosed were observed with oral pretomanid during organogenesis at exposures up to approximately two times the approved clinical dose. Pretomanid was not teratogenic or a selective developmental toxicant at exposures up to approximately four times the clinical exposure at the approved clinical dose of pretomanid. In rats, post-implantation loss and lower fetal body weights were observed only in the presence of maternal toxicity.

In the PPND study, there were no adverse developmental effects in pups of pregnant rats orally dosed with pretomanid throughout gestation and lactation at exposures up to approximately 1.1 times the approved clinical dose of pretomanid. Maternal exposure to pretomanid throughout gestation and lactation did not affect pup survival, post-weaning growth, or otherwise affect offspring development at any dose level. At maternally toxic doses (approximately three times the approved clinical dose), reduced pup body weights during lactation (up to postnatal day 21) and a delay in attainment of the righting reflex were observed, but the body weights rebounded following cessation of exposure from nursing and during post-weaning maturation. Importantly, there were no effects on fertility in the F1 generation exposed *in utero* and throughout lactation at any dose level, as was seen in the FEED study in adult male rats dosed directly. Pretomanid was transferred to breast milk at all dose levels, and the concentration was higher in milk than plasma; however, pup plasma concentrations were much lower than maternal plasma concentrations.

### Interpretation of male reproductive findings

Based on the EFD and PPND data in rats, the testicular toxicity of pretomanid observed in general toxicity and fertility studies, which were conducted in near or fully sexually mature rats, does not appear to be due to an effect on male reproductive development during *in utero* or lactational exposures. No malformations in the reproductive tract were observed in EFD studies in rats and rabbits, where fetal exposures were similar to exposures achieved in maternal animals directly dosed with pretomanid. In addition, no effects on fertility were observed in rats that were exposed to pretomanid via maternal dosing throughout gestation and lactation in the PPND study. It is likely that fetal exposures remained consistent between fetuses and maternal animals throughout gestation during the PPND study (although not measured); however, during lactation, plasma concentrations of pretomanid in pups were lower than plasma concentrations in maternal animals, indicating that systemic exposure in pups was not sustained during lactation.

This supports that pretomanid has a lower risk of impacting testicular development and function in male fetuses and neonates following maternal exposure during pregnancy and lactation.

### Context within TB drug development and regulatory frameworks

Many of the TB drugs used in pregnant and lactating women are old drugs with limited clinical and nonclinical developmental and reproductive data and/or complex safety profiles. Nearly all older TB drugs were given the historical classification of Pregnancy Category C, indicating evidence of fetal risk in animals but insufficient well-controlled studies in humans (19). Under current ICH S5(R3) guidance, increased concern is warranted when the NOAELs in nonclinical development and reproductive toxicology studies occur at exposures less than 10-fold the human exposure at the approved clinical dose. Low reproductive safety margins relative to clinical exposure are a common challenge for antibiotics (12) and are particularly common for TB drugs, which are often dosed near the upper limit of tolerability to maximize efficacy and shorten treatment duration.

Within this context, pretomanid has a similar nonclinical benefit-risk profile for pregnant and lactating women as delamanid (Table 7), which is another nitroimidazole used to treat MDR-TB and is currently recommended for use in pregnant and lactating women by the WHO in the BDLLfxZ regimen. The only difference between the two drugs in the European Medicines Agency (EMA) Summary of Product Characteristics (SmPC) relates to testicular toxicity identified in adult male rats with pretomanid (20, 21). It is notable that the testicular toxicity identified in rodent studies with pretomanid has not been identified in male monkey repeat-dose toxicology studies (16), in humans in a study dedicated specifically to evaluate semen parameters in 22 adult males with DR-TB and treated with BPaMZ for 6 months (22), after a male hormone analysis of four clinical trials with pretomanid-containing regimens (23), or in a paternity survey from males who participated in the STAND, SimpliciTB Nix, and Zenix clinical trials (22). In the 2022 operational handbook on tuberculosis treatment for MDR-TB, the WHO considers the latter evidence sufficient to address concerns of testicular toxicity in humans (24).

**TABLE 7:** Summary of preclinical development and reproductive toxicology data from EMA summary of product characteristics

Drug	Summary of Product Characteristics (Section 4.6)	Lay summary from label
Pretomanid	SPC (23 June 2023) Pregnancy: There are very limited amounts of data from the use of pretomanid in pregnant women. Animal studies do not indicate direct or indirect harmful effects with respect to embryo-fetal development. Pretomanid should be used during pregnancy only if the benefit to the patient is considered to outweigh the potential risk to the fetus. Lactation: It is unknown whether pretomanid/metabolites are excreted in human milk. Available pharmacodynamic/toxicological data in animals have shown excretion of pretomanid in milk. A risk to the suckling child cannot be excluded. A decision must be made whether to discontinue breastfeeding or pretomanid therapy, taking into account the benefit of breastfeeding for the child and the benefit of therapy for the woman.	Fertility and early embryonic development study. Testicular toxicity without exposure margin to the approved clinical dose of pretomanid.
		Embryofetal development studies. No effect in animals. Use in pregnancy only if benefit outweighs potential risk.
		Pre- and postnatal development study. Excretion of the drug in the milk of rats. A benefit:risk decision must be made on continuing therapy in lactation.
Delamanid	SPC (24 March 2023) Pregnancy: There are no or limited amounts of data from the use of delamanid in pregnant women. Studies in animals have shown reproductive toxicity. Deltyba is not recommended during pregnancy and in women of childbearing potential not using contraception. Lactation: It is unknown whether delamanid/metabolites are excreted in human milk. Available pharmacokinetic/toxicological data in animals have shown excretion of delamanid and/or its metabolites in milk. A risk to the newborns/infants cannot be excluded. It is recommended that women should not breastfeed during treatment with Deltyba.	Fertility and early embryonic development study. No effect.
		Embryofetal development study. Embryofetal toxicity in rabbits at a maternally toxic dose. Not recommended in pregnancy.
		Pre- and postnatal development study. Excretion of the drug in the milk of rats. Recommendation for women not to breastfeed during treatment.

### Regulatory labeling and clinical decision-making

Despite delamanid being recommended for use in pregnant and lactating women by the WHO at the time of this publication, the EMA SmPC for delamanid states that it is not recommended to be used in pregnant and lactating women, while the EMA summary of product characteristics for pretomanid states that it is recommended to be used if the benefits outweigh the risks. These discrepancies highlight the complexity clinicians face when navigating differing regulatory and public health recommendations, particularly in settings with limited data.

### Available clinical pregnancy data and remaining gaps

Clinical experience with pretomanid during pregnancy remains limited, and data in lactating women are currently unavailable. In pretomanid clinical studies to date, identification of pregnancy during treatment generally led to discontinuation of study drugs in accordance with protocol requirements. Identification of pregnancy at or shortly after the end of treatment provides an opportunity for data collection in women who elected to continue gestation. A search of the TB Alliance safety database identified six such cases: five resulted in live births without reported congenital anomalies, and one resulted in spontaneous abortion. Interpretation of these findings is constrained by the small sample size and the background risk of early pregnancy loss, which is estimated to be up to approximately 20% in the general population (25). One point the authors want to emphasize is the insufficient availability of case-level data to support meaningful assessment of potential drug-related risks to mother and unborn child. Key information—such as gestational age at exposure, timing and duration of drug exposure, maternal clinical characteristics, pregnancy history, neonatal outcomes, timing of spontaneous abortions, and longitudinal follow-up of infants for growth and neurodevelopment—was incomplete or unavailable. This experience underscores a critical gap: pregnancies occurring during or shortly after the end of treatment in clinical trials represent important opportunities for systematic data collection to inform the safety profile of investigational agents (and regimens), and the data included in the general pregnancy-case report forms during clinical trials should be enhanced.

To date, no anti-TB drugs have been studied in large, dedicated, prospective clinical trials specifically designed to assess efficacy and safety in pregnant or lactating women with TB disease (6, 18). Nonetheless, several agents, including bedaquiline and linezolid, are recommended by WHO for use in these populations based on limited human data, case reports, and risk-benefit considerations (24, 26).

In this context, systematic, prospective, and standardized data collection in women who become pregnant during clinical trials is essential to incrementally build evidence that can inform clinical and regulatory decision-making until dedicated studies are available. At a minimum, the resulting data could inform decisions to conduct clinical trials in pregnant and lactating women by influencing the balance of risk and benefit. This more patient-focused approach has the potential to accelerate the generation of evidence in the context of well-controlled trials, resulting in meaningful labeling information for prescribers and patients over a considerably shorter time period than postmarket surveillance, which can take decades.

### Conclusions

Taken together, the weight of evidence from comprehensive nonclinical reproductive and developmental toxicology studies and limited clinical experience supports further evaluation of pretomanid for use in pregnant and lactating women, particularly given the urgent need for shorter and more effective RR/MDR-TB treatment regimens in these populations. In addition, the limited scope of pregnancy-related data routinely captured in TB clinical trials and during postmarket surveillance highlights the need for more comprehensive collection of maternal, pregnancy, and infant outcomes to accelerate the understanding of the safety and efficacy of pretomanid-containing regimens.

## SUPPLEMENTAL MATERIAL

Table S1 aac.00343-26-s0001.docx List of completed or ongoing clinical trials with pretomanid where pregnancies were reported.

ASM does not own the copyrights to Supplemental Material that may be linked to, or accessed through, an article. The authors have granted ASM a non-exclusive, world-wide license to publish the Supplemental Material files. Please contact the corresponding author directly for reuse.
