# Supplementary material for: Safety evaluation of pretomanid, an anti-tuberculosis drug, for the treatment of pregnant and lactating women
Source: Antimicrob Agents Chemother. 2026 Jul 10;70(8):e00343-26. doi: 10.1128/aac.00343-26 (PMC13435736; doi:10.1128/aac.00343-26)
Supplement: Supplementary file 1 [file aac.00343-26-s0001.docx]

**Supplementary Table 1.** List of Completed or Ongoing Clinical Trials Where Pregnancies Were Reported

| **Study Identifier** | **Objectives of the Study** | **Study Design** | **Test Products;** **Dosage Regimen** | **Number of Participants Enrolled/ Number of pregnancies*** | **Duration of Treatment with Pretomanid/ Timing of the pregnancy tests** |
| --- | --- | --- | --- | --- | --- |
| DMID 10‑0058  (Clinicaltrials.gov: NCT01674218) | Safety (cardiac repolarization [QT/QTc]) PK and tolerability | Phase 1 double-blind‑, randomized, crossover (5‑period, 5‑treatment), placebo-controlled | Pretomanid, moxifloxacin  Single dose  400 mg or 1000 mg pretomanid alone  Or  400 mg pretomind + 400 mg moxifloxacin | 74/**1** | Single dose; 33 days (total)  Pregnancy tests were done throughout the study including the last day of treatment. (baseline, at each period and end of the study) |
| NA_00093014  (Clinicaltrials.gov: NCT02256696) | Efficacy, safety | Phase 2 randomized,  open-label trial of PA-824-containing regimens versus standard treatment for drug-sensitive TB | Pretomanid, HRZ  PaHRZ for the first 8 weeks then PaHR from week 9 to week 12 then HR from week 13 to week 24 | 157/**2** | 24 weeks  Pregnancy tests were done throughout the study including the last day of treatment |
| NC-005  (Clinicaltrials.gov: NCT02193776) | Efficacy, safety, tolerability, and PK | Phase 2b partially randomized, open-label, parallel-groups controlled-HRZE | Bedaquiline, pretomanid, pyrazinamide, HRZE, moxifloxacin;  **DS-TB participants**:  BPaZ: bedaquiline 200 mg, pretomanid 200 mg, and pyrazinamide 1500 mg  BPaZ: bedaquiline 400 mg days 1-14 and 200 mg 3 times/week days 15-56, pretomanid 200 mg, and pyrazinamide 1500 mg  HRZE  **MDR-TB participants:**  BPaMZ: bedaquiline 200 mg, pretomanid 200 mg, moxifloxicin 400 mg and pyrazinamide 1500 mg | 240/**2** | 56 days  Pregnancy tests were done throughout the study including the last day of treatment (baseline, days 29, 57 and 70) |
| STAND/ NC-006  (Clinicaltrials.gov: NCT02342886) | Efficacy, safety, and tolerability | Phase 3 partially randomized, open-label, parallel-group  Controlled-HRZE/HR | Pretomanid, moxifloxacin, pyrazinamide, HRZE, HR;  **DS-TB:**  PaMZ: Pretomanid 100 mg; moxifloxacin 400 mg; pyrazinamide 1500 mg  PaMZ: Pretomanid 200 mg, moxifloxacin 400 mg, pyrazinamide 1500 mg  HRZE/HR  **MDR-TB**:  PaMZ: pretomanid 200 mg, moxifloxacin 400 mg, pyrazinamide 1500 mg | 284/**4** | 17 or 26 weeks / Pregnancy tests were done throughout the study: baseline, weeks 8, 17 and 26 (for those randomized to 26 weeks of treatment) |
| Nix-TB  (Clinicaltrials.gov: NCT02333799) | Efficacy, safety, tolerability, and PK | Phase 3 open-label, uncontrolled | Bedaquiline, pretomanid, linezolid;  BPaL: bedaquiline 400 mg D1‑14 and then 200 mg 3 times/week; pretomanid 200 mg, linezolid 1200 mg | 109**/6** | 26 weeks (up to 39 weeks in participants who were culture positive or reverted to being culture positive between Month 4 and Month 6).  Pregnancy tests were done throughout the study including the last day of treatment (baseline, week 8 and 26) |
| ZeNix/  NC-007  (Clinicaltrials.gov: NCT03086486) | Efficacy PK, safety, and tolerability | Phase 3 randomized, partially-blinded parallel-group  Controlled | Bedaquiline, pretomanid, linezolid;  BPaL: bedaquiline 200 mg for 8 weeks, then 100 mg for 18 weeks; pretomanid 200 mg; linezolid 1200 mg  BPaL: bedaquiline 200 mg for 8 weeks, then 100 mg for 18 weeks; pretomanid 200 mg; linezolid 1200 mg for 9 weeks, then placebo for 17 weeks  BPaL: bedaquiline 200 mg for 8 weeks, then 100 mg for 18 weeks; pretomanid 200 mg; linezolid 600 mg  BPaL: bedaquiline 200 mg for 8 weeks, then 100 mg for 18 weeks; pretomanid 200 mg; linezolid 600 mg for 9 weeks, then placebo for 17 weeks | 181**/4** | 26 weeks  Pregnancy tests were done throughout the including the last day of treatment study (baseline, weeks 8, 16 and EOT) |
| SimpliciTB/ NC-008*  (Clinicaltrials.gov: NCT03338621) | Efficacy and safety | Phase 2c open-label, partially randomized  Controlled-HRZE/HR | Bedaquiline, pretomanid, moxifloxacin, pyrazinamide; Oral; Once daily;  **DS-TB**  BPaMZ: bedaquiline 200 mg for 8 weeks then 100 mg for 9 weeks; pretomanid 200 mg; moxifloxacin 400 mg; pyrazinamide 1500 mg  HRZE/HR  **DR-TB**  BPaMZ: bedaquiline 200 mg for 8 weeks then 100 mg for 18 weeks; pretomanid 200 mg; moxifloxacin 400 mg; pyrazinamide 1500 mg | 452  (299 BPaMZ arms and 153 HRZE)/**6** | 17 to 26 weeks, depending on the treatment  Pregnancy tests were done throughout the study including the last day of treatment (baseline, weeks 2, 8, 17 and 26) |
| TB Practecal  (Clinicaltrials.gov: NCT02589782 and reported in Crocker-Buque et al, 2024) | Efficacy and safety | Phase II–III multi-centre controlled, open-label, randomized trial | Bedaquiline, pretomanid, moxifloxacin, linezolid, clofazimine  BPaML: bedaquiline 400 mg for 8 weeks then 200 mg for 22 weeks; pretomanid 200 mg; moxifloxacin 400 mg; for 24 weeks, linezolid 600 mg 16 weeks followed by 300 mg 8 weeks (or 600 mg 3 times per week)  BPaLC: Bedaquiline 400 mg for 8 weeks then 200 mg for 22 weeks; pretomanid 200 mg linezolid 600 mg 16 weeks followed by 300 mg 8 weeks (or 600 mg 3times per week), clofazimine 50 mg (≤ 33 kg) or 100 mg (> 33 kg for 24 weeks)  BPaL: bedaquiline 400 mg for 8 weeks then 200 mg for 22 weeks; pretomanid 200 mg; linezolid 600 mg 16 weeks followed by 300 mg 8 weeks (or 600 mg 3 times per week)  HRZE/HR | 552  (BPamL 151, BPaLC, 126  BPaL 123,  HRZE 152**)/16** | 24 weeks  Pregnancy tests were done at screening and through the study (baseline and Weeks 12, 16, 20 and 24). |
| ICMR-National Institute for Research in Tuberculosis  mBPaL  (Clinicaltrials.gov: NCT03086486) | Efficacy, safety and tolerability | Evaluate the effectiveness, safety, and tolerability of various doses of Linezolid in combination with Bedaquiline and Pretomanid | Arm 1: 26 weeks of bedaquiline, pretomanid and linezolid (600mg)  Arm 2: 9 weeks bedaquiline, pretomanid and linezolid (600mg) followed by 17 weeks bedaquiline, pretomanid and linezolid (300mg)  Arm 3: 13 weeks of bedaquiline, pretomanid and linezolid (600mg) followed by 13 weeks bedaquiline, pretomanid and linezolid (300mg) | 403/**2** | 26 weeks/39 weeks in case of culture positivity at 16th week  Pregnancy tests were done throughout the study including the last day of treatment |
| Pan-TB (ongoing)  (Clinicaltrials.gov: NCT05686356) | Efficacy and safety | A Pan-TB Regimen Targeting Host and Microbe (panTB-HM) to evaluate different regimens of TB medications | Arm 1 (S1200BP)  Sutezolid 1200mg QD plus bedaquiline and pretomanid for 4 months  Arm 2 (S1600BP)  Sutezolid 1600mg QD plus bedaquiline and pretomanid for 4 months  Arm 3 (S1600BPN)  Sutezolid 1600mg QD plus bedaquiline pretomanid and N-acetyl cysteine for 4 months  Arm 4 (HRZE)  Rifafour (2HRZE/4HR) | 92/**1** | 4 months  Pregnancy tests were done throughout the study (baseline, weeks 12 and 24) |

Abbreviations: H = isoniazid; R = rifampin, E = ethambutol; Z = pyrazinamide; B = bedaquiline, Pa or PA-824 = pretomanid; C = Clofazimine; S = sutezolid; M = moxifloxacin, L = linezolid; TB = tuberculosis; PK = pharmacokinetic; DS-TB = drug sensitive tuberculosis; MDR-TB = multi-drug resistant tuberculosis;

*Only pregnancies occurring from the first day of treatment up to 30 days after the end of treatment with pretomanid were discussed in this manuscript.

**Reference**

1) Crocker-Buque T, Lachenal N, Narasimooloo C, Abdrasuliev T, Parpieva N, Tigay Z, Liverko I, Usmanova R, Butabekov I, Moodliar R, Mbenga M, Rasool M, Nyang'wa BT, Berry C. 2024. Pregnancy Outcomes in Multidrug-Resistant Tuberculosis in TB-PRACTECAL. *Clin Infect Dis* 79:569-570.
